# Updates on renal phosphate transport

**DOI:** 10.1097/MNH.0000000000001090

**Published:** 2025-05-13

**Authors:** Carsten Alexande Wagner, Daniela Egli-Spichtig, Isabel Rubio-Aliaga

**Affiliations:** Institute of Physiology and Zurich Kidney Center, University of Zurich, Zurich, Switzerland

**Keywords:** fibroblast growth factor 23, nephrolithiasis, parathyroid hormone, phosphate transporters

## Abstract

**Purpose of review:**

The kidneys control systemic phosphate balance by regulating phosphate transporters mediating the reabsorption of inorganic phosphate (Pi). At least three different Na^+^-driven Pi cotransporters are located in the brush border membrane (BBM) of proximal tubule cells, NaPi-IIa (SLC34A1), NaPi-IIc (SLC34A3) and PiT-2 (SLC20A2). This review will discuss novel aspects of their regulation, pharmacology, and genetics.

**Recent findings:**

Renal NaPi transporters are not only acutely regulated by the phosphaturic hormones parathyroid hormone (PTH) and Fibroblast Growth Factor 23 (FGF23) but possibly also by further mechanisms. A role of inositol hexakisphosphate (IP6) kinases has been found and their deletion from kidneys causes hypophosphatemia, hyperphosphaturia, and bone demineralization. Inhibitors of NaPis elicit phosphaturia and may reduce levels of PTH and FGF23 in chronic kidney disease (CKD) models. The relevance of renal NaPi transporters is highlighted by loss-of-function mutations in SLC34 transporters and analysis of patients provides new insights into diseases caused by variants. Major manifestations include nephrocalcinosis and -lithiasis, rickets, and variants may predispose to an accelerated decline in kidney function.

**Summary:**

Renal Pi transporters are regulated, may provide novel drug targets for prevention or treatment of hyperphosphatemia, and contribute to the genetic risk to develop kidney stones and CKD.

## INTRODUCTION

Renal filtration, reabsorption and excretion of inorganic phosphate ions (H_2_PO_4_^−^/HPO_4_^2−^; abbreviated as Pi) is a major determinant of whole-body Pi homeostasis, which is critical for normal cellular and organ function [1–3]. Intestinal absorption of Pi is only partially regulated [4], making the kidneys the body's gatekeepers of phosphate balance. Several pathophysiological conditions and genetic diseases caused by or associated with altered renal reabsorption of Pi have been described, underscoring the importance of this process [2,3,5–7]. The basic characteristics of epithelial phosphate transport, of renal Pi handling, and the regulation of renal Pi excretion have been reviewed in a number of articles [8–11].

In this review, we discuss novel aspects of the regulation and pharmacology of the sodium-dependent Pi co-transporters responsible for renal Pi reabsorption. Furthermore, recent data highlight the relevance of genetic variants in the genes encoding NaPi-IIa (SLC34A1) and NaPi-IIc (SC34A3) in pediatric patients with Infantile idiopathic hypercalcemia or Hereditary Hypophosphatemic Rickets with Hypercalcuria (HHRH), and in adults with kidney stones as well as in patients with chronic kidney disease (CKD). 

**Box 1:**
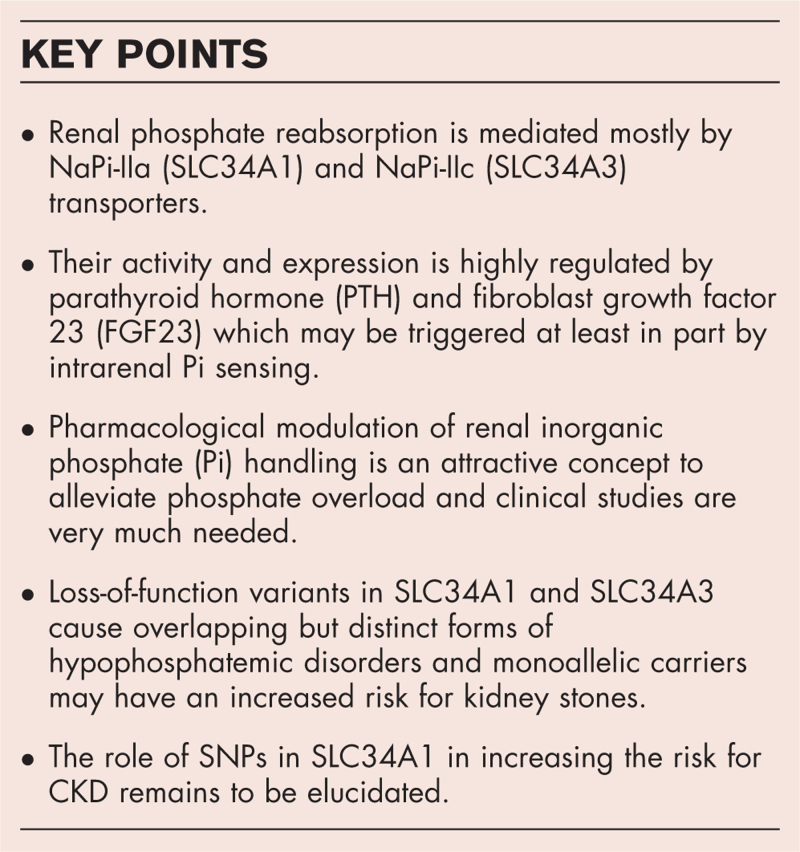
no caption available

## PROXIMAL TUBULAR REABSORPTION OF PHOSPHATE

Under conditions of normal dietary Pi intake and kidney function, approximately 80% of filtered Pi is reabsorbed in the renal proximal tubules [12]. There is no functional evidence for Pi reabsorption in the segments of the loop of Henle, and Pi reabsorption in the distal part of the nephron remains to be elucidated [13,14]. Taking into account different methodological approaches and species differences, it appears that up to 10% of the filtered load may be handled by distal tubular segments, but the molecular mechanisms remain to be defined.

In the proximal tubule, the apical reabsorption of Pi is strictly dependent on the presence of sodium ions (i.e. occurs via secondary active sodium-dependent transport mechanism(s): Na/Pi cotransport). In brush border membrane vesicles, the overall Na/Pi cotransport activity is modulated by extracellular pH, with higher uptake rates at more alkaline pH [15].

**FIGURE 1:**
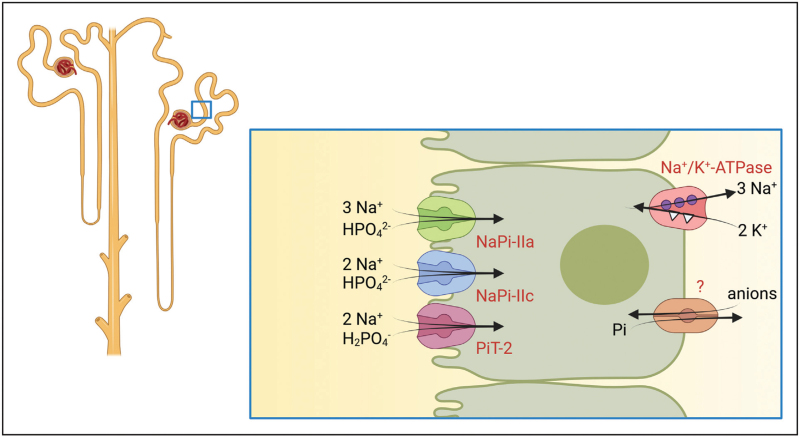
Na^+^-dependent phosphate transporters localized in the proximal tubule. Proximal tubule cells express at least three distinct Na^+^-dependent Pi transporters in their luminal membrane, NaPi-IIa, NaPi-IIc and PiT-2, that mediate the initial uptake of Na^+^ and Pi with different stoichiometries and specific preferences for mono- vs divalent Pi. The basolateral efflux mechanism for Pi is unknown and might involve the exchange of Pi versus an extracellular anion. The apical entry step of Pi is energized by basolateral Na^+^/K^+^-ATPases that maintain low intracellular Na^+^ concentrations and a negative membrane potential.

Functional studies, rodent studies, and human genetics collectively support an important role for SLC20 and SLC34 family members in Pi transport. Current evidence suggests that the SLC34 Na/Pi transporters NaPi-IIa (SLC34A1) and NaPi-IIc (SLC34A3) mediate the majority of renal Pi reabsorption, whereas the roles of NaPi-IIb (SLC34A2), Pit-1 (SLC20A1) and Pit-2 (SLC20A2) in the kidney are less clear. Both NaPi-IIa and NaPi-IIc are highly expressed in the brush border membrane of the early proximal tubule (Fig. 1) [16–18]. Additionally, Pit-2 has also been localized to this nephron segment [19]. In contrast, NaPi-IIb has recently been detected in the loop of Henle, but without functional data [20], although its main sites of expression are lungs, small intestine, and salivary glands [8,21]. As discussed below, genetic evidence from transgenic animal models indicates that in rodents NaPi-IIa mediates up to 80% of the Na^+^-dependent Pi uptake in the proximal tubule [22,23]. NaPi-IIc does not appear to play a major role in the adult rodent kidney [24,25], but may be more important in the early postnatal period and in weaning animals [26].

The mechanisms by which Pi exits at the basolateral side of the proximal tubular cell are not well defined and the corresponding Pi transporter proteins have not been identified. Based on studies in opossum kidney (OK) cells, several mechanisms for the basolateral exit of Pi have been proposed, such as a phosphate/anion (bicarbonate) exchange or a sodium-independent, pathway [27–29]. Recent studies propose XPR1 (Xenotropic and polytropic retrovirus receptor 1) as a candidate protein. Its conditional genetic deletion in renal tubules in mice caused a Fanconi-like syndrome [30], while patients carrying inactivating monoallelic mutations suffer from basal ganglia calcification with no renal phenotype reported to date [31]. The subcellular localization of XPR1 in mammalian cells remains unknown.

## REGULATION OF PROXIMAL TUBULAR REABSORPTION BY PHOSPHATURIC HORMONES AND INORGANIC PHOSPHATE

**FIGURE 2:**
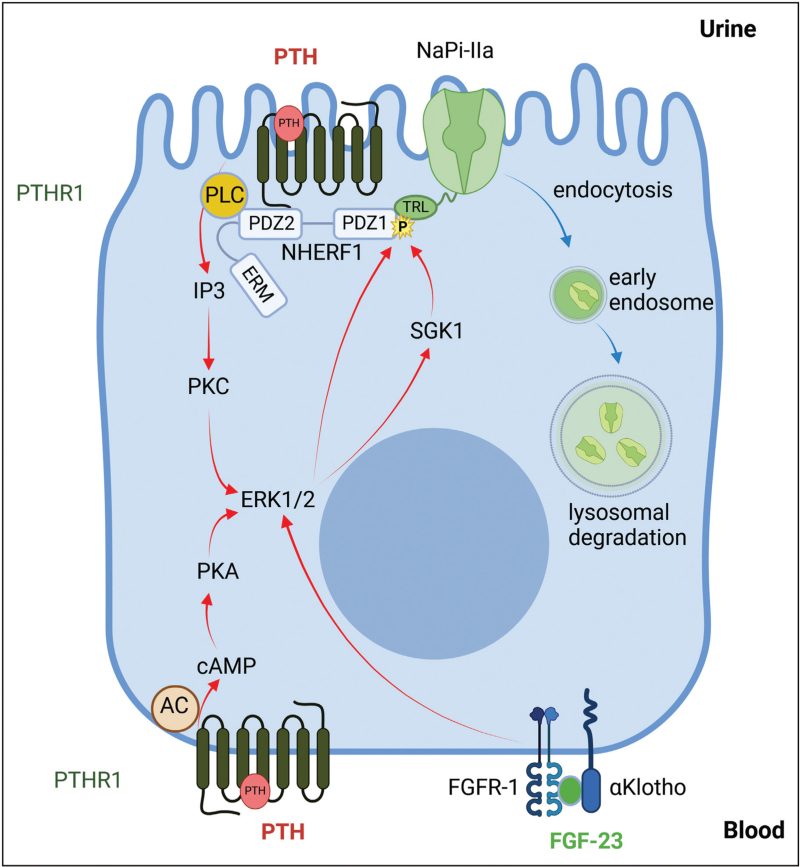
Regulation of NaPi-IIa and NaPi-IIc transporters by PTH and FGF23 induced signaling in renal proximal tubules. Binding of PTH to its receptor (PTHR1) can activate either the phospholipase C (PLC) or the adenylate cyclase (AC) pathways, leading to stimulation of PKC and PKA protein kinases, respectively. Both pathways partially converge at the level of the MAPK-kinase ERK1/2, and lead to phosphorylation of NHERF1 destabilizing the association between the scaffolding protein and NaPi-IIa; this destabilization allows the endocytosis of the transporter and lysosomal degradation of NaPi-IIa. In the presence of aklotho, binding of FGF-23 to FGFR1 also activates ERK1/2 and triggers phosphorylation of NHERF1, with subsequent internalization and degradation of NaPi-IIa.

Because renal Pi excretion is the gatekeeper of systemic Pi homeostasis, a variety of factors modulate Pi reabsorption. Current evidence suggests that changes in the rate of Pi reabsorption are mostly mediated by changes in NaPi-IIa and NaPi-IIc protein abundance rather than by regulation of transporter activity [12]. NaPi-IIa and NaPi-IIc downregulation is mainly mediated by PTH, FGF23, dopamine, atrial natriuretic hormone (ANP), and acidosis, while thyroid hormone and growth hormone stimulate their expression [8,12]. High dietary Pi intake causes phosphaturia by downregulating NaPi-IIa and NaPi-IIc. This effect is in part mediated by PTH and FGF23 (Fig. 2), albeit with a distinct time course [32]. In rodents and humans, acute phosphate loading increases blood Pi and PTH levels within minutes while the increase in FGF23 takes 3–4 h [33,34]. With chronic Pi loading, PTH appears to return to normal levels while FGF23 remains elevated [35]. PTH and FGF23 stimulate the internalization of NaPi-IIa and NaPi-IIc leading to their routing to lysosomes [36,37]. In rats, acute parathyroidectomy reduces phosphaturia in response to Pi loading [32], whereas in mice genetic deletion of PTH has little effect on phosphaturia, suggesting that additional factors compensate the absence of PTH and induce phosphaturia [33]. Blocking FGF23 signaling in the absence of PTH also has no effect on the acute phosphaturia and no increase in dopamine or PTH related peptide (PTHrP) was observed [33]. The calcium-sensing receptor (CaSR) binds Pi, rendering the receptor less sensitive to extracellular calcium [38^▪▪^]. In the parathyroid glands, this results in increased secretion of PTH in response to an increase in extracellular Pi. Whether functional CaSR is expressed in the proximal tubule has remained controversial. Recent studies modulating CaSR activity by genetic and pharmacological manipulations, showed that CaSR signaling is critical for the immediate response of PTH to Pi loading but does not directly induce phosphaturia [39,40].

Inositol hexakisphosphate (IP6) kinases (IP6Ks) are involved in Pi sensing in yeast and in some bacteria and are also present in mammalian cells [41,42]. They mediate the synthesis of 5-IP7 from IP6 and of 1,5-IP8 from 1-IP7. Kidneys express two of the three paralogues, IP6K1 and IP6K2. In OK cells, a cell model for the proximal tubule, inhibition or genetic ablation of both kinases reduced Na^+^-dependent Pi transport and its adaptation to ambient Pi concentrations [43^▪▪^]. Conditional deletion of both kinases in kidney epithelial cells in mice, strongly reduced 5-IP7 and 1,5IP8 levels and NaPi-IIa and NaPi-IIc were almost absent both at mRNA and protein levels, causing hypophosphatemia and reduced Pi transport activity in isolated brush border membrane vesicles. Hypophosphatemia also resulted in lower FGF23 levels and reduced bone mineral density. However, the effect of reduced polyphosphate signaling was not specific to renal Pi handling. Several transport pathways in the proximal tubule were affected, as evidenced by reduced expression of proximal tubule glucose transporters, reduced expression of the endocytic receptor megalin and albuminuria, and reduced expression of the Na/H exchanger 3 (NHE3) and claudin 2, leading to hypercalciuria.

Recently, in renal disease it has been proposed that NaPi-IIa-mediated Pi uptake regulates glycolysis in the renal proximal tubules and eventually glycerol-3-phosphate (G3P) production, which in turn regulates FGF23 expression and secretion in bone [44,45^▪▪^]. Whether this mechanism is also relevant in physiological conditions and CKD needs further investigation.

## INHIBITION OF PROXIMAL TUBULE INORGANIC PHOSPHATE REABSORPTION TO PREVENT HYPERPHOSPHATEMIA IN KIDNEY DISEASE

Both acute kidney injury (AKI) and CKD may cause hyperphosphatemia [2,46,47]. A recent study in mice suggests that normal to high phosphate intake may contribute to AKI-induced morbidity and mortality [48^▪^]. In patients with CKD-mineral bone disorder (MBD) poor control of phosphate levels is associated with poor prognosis [6]. Current strategies to improve CKD-MBD target either intestinal absorption of Pi with Pi binders, reduced dietary Pi consumption or reduce intestinal Pi absorption with tenapanor while other therapies aim to reduce hyperparathyroidism and to improve calcitriol deficiency [6]. Tenapanor, a nonabsorbable NHE3 inhibitor, may reduce paracellular Pi absorption by inducing intracellular acidification that may decrease paracellular Pi permeability [49]. Novel therapies may include reducing intestinal Pi absorption by using pan-blockers of intestinal Pi transporters or by increasing renal Pi excretion by inhibiting NaPi-IIa [50]. This latter approach would increase renal Pi clearance in earlier stages of CKD when renal filtration rates are sufficient to filter relevant amounts of Pi. Two compounds have been reported to specifically block renal NaPi-IIa transporters. One compound, PF-06869206, is an orally active inhibitor of NaPi-IIa with an IC_50_ of about 0.4 μmol/l [51]. This compound has been used by two different groups in mice and rats [52^▪^,53^▪^,^54^]. PF-06869206 acutely induced phosphaturia, calciuria and increased NaCl excretion while other solutes such as glucose or amino acids were not affected. Stimulated phosphaturia lowers plasma Pi levels in normal mice. In one mouse model but not in another, PTH and FGF23 levels were reduced [52^▪^,53^▪^]. In mice and rats with 5/6 nephrectomy, PF-06869206 also induced phosphaturia but to a much lesser extent possibly reflecting lower nephron mass and downregulation of NaPi-IIa in CKD [52^▪^,53^▪^]. The natriuretic effect is preserved in NaPi-IIa-deficient mice suggesting off-target effects on the epithelial sodium channel ENaC [54]. Another compound, BAY 767, selectively inhibited rat and human NaPi-IIa paralogues with an apparent Ki ∼3–6 nmol/l [55] and stimulated renal Pi excretion in normal rats. In a rat model with induced vascular calcification, BAY 767 reduced hyperphosphatemia and vascular calcifications together with PTH and FGF23.

Human data are lacking for both compounds. Moreover, as genetic data suggest a relevant contribution of NaPi-IIc to renal Pi handling in adults, inhibitors targeting NaPi-IIa and NaPi-IIc may be required to achieve sufficient inhibition. Also, the regulation of renal Pi transporters in human CKD remains largely unknown and needs to be addressed.

## GENETICS OF RENAL PHOSPHATE TRANSPORTERS

As discussed above, studies in mouse models indicated that the majority of renal reabsorption of Pi in adult rodent is mediated by NaPi-IIa, whereas the contribution of NaPi-IIc is negligible. Human genetics, however, suggest a different and more complex picture. Loss of function genetic variants in both SLC34A1 and SLC34A3 have been reported in patients with idiopathic infantile hypercalcemia (IIH) [56,57] and Hereditary Hypophosphatemic Rickets with Hypercalcuria (HHRH) [58,59]. IIH patients with biallelic SLC34A1 loss of function variants present mostly with hyperphosphaturia, hypophosphatemia, and subsequently elevated calcitriol levels with hypercalcemia, hypercalcuria, and nephrocalcinosis or -lithiasis [57]. Despite that HHRH patients develop similar manifestations, a few distinct differences exist. HHRH patients often suffer from rickets which is not observed in IIH patients with SLC34A1 variants. Moreover, HHRH persists into adulthood while it appears that disease symptoms may improve in IIH patients with SLC34A1 variants. The role of monoallelic variants in both genes also had remained unclear. Recent detailed reports of a large number of kindreds and individuals with either SLC34A1 or SLC34A3 variants have shed new light on these questions [60^▪▪^,^61^^▪▪^]. First, these studies provide a description of typical features associated with the genetic status of biallelic and monoallelic carriers. Carriers of biallelic SLC34A1 variants had often hypophosphatemia, hyperphosphaturia (or decreased TmP/GFR or TRP), elevated calcitriol, and hypercalciuria with failure to thrive, bone manifestations, and nephrocalcinosis/-lithiasis. The latter, was also present in many monoallelic carriers of SLC34A1 variants suggesting a relevant risk of monoallelic carriers to develop kidney calcifications [60^▪▪^]. In the case of SLC34A3 variants, a majority of patients has hypophosphatemia, hyperphosphaturia, elevated calcitriol, hypercalcemia, hypercalciuria, and kidney stones or nephrocalcinosis together with a bone manifestation but a relevant fraction of patients has only either kidney or bone pathologies [60^▪▪^,^61^^▪▪^]. Importantly, biallelic carriers of SLC34A3 variants appear to have an increased risk for CKD in adulthood [60^▪▪^]. For patients with biallelic SLC34A1 variants no long-term follow up data are available to assess eGFR at later ages but case reports indicate that at least some patients do develop CKD in early adulthood [62].

Second, disease caused by variants in SLC34A1 and SLC34A3 has been classically considered as autosomal recessive disorders. However, many SLC34A1 and SLC34A3 monoallelic carriers appear to have a lower TmP/GFR, elevated calcitriol levels, hypercalciuria, and a higher risk to develop kidney stones than the general population [61^▪▪^,^62^]. One of the most frequent pathogenic variants in SLC34A3 is the missense mutation Ser192Leu which was found in a large population based health record data base in 0.1% of individuals. The monoallelic carriers had an elevated risk for kidney stones, hypophosphatemia, and lower eGFR [63]. The findings in monoallelic carriers of SLC34A1 or SLC34A3 variants may be clinically highly relevant as up to 4–5% of the general population may carry such variants. However, whether all variants are pathogenic remains to be clarified.

Genome wide association studies (GWAS) identified over 100 loci that associate with the risk for eGFR decline and CKD across all major ethnic groups [64–66,67^▪▪^]. Among the loci frequently identified is SLC34A1 and single nucleotide polymorphisms (SNPs) in this region associate with lower expression of SLC34A1 mRNA and SLC34A1 mRNA abundance is lower in CKD kidneys [68]. Whether the reduction of NaPi-IIa expression is causal for the increased risk to develop CKD remains to be established. Notably, some patients with loss-of-function variants in SLC34A1 progressed early to CKD which has been attributed to nephrocalcinosis and kidney stones [62].

Beyond the clinical relevance of these findings, the age-dependent relevance of NaPi-IIa and NaPi-IIc for renal Pi handling might be species dependent. While rodent data clearly demonstrate a brief postnatal period with a role of NaPi-IIc, and the predominant role of NaPi-IIa in adult mice and rats, in humans NaPi-IIa and NaPi-IIc appear to have both relevance during infancy and a persistently important role of NaPi-IIc in adult kidney. Further data are required to address this important question which is highly relevant for future attempts to pharmacologically target renal Pi transport.

## CONCLUSION

Renal Pi handling is highly regulated and novel links between renal Pi-sensing and regulation of FGF23 are emerging highlighting the intricate interactions between bone and kidney. IP6Ks may contribute to intracellular Pi sensing in renal tubular cells but their role clearly goes beyond direct control of renal Pi handling. The critical role of renal Pi handling for systemic Pi balance as evident from rodent studies and inherent or acquired diseases in humans directly or indirectly affecting renal Pi transporters such as in patients with SLC34A1 or SLC34A3 variants, or in patients with inherited or acquired forms of altered FGF23 or PTH levels. Both biallelic and monoallelic loss of function variants can cause disease with distinct differences between SLC34A1 and SLC34A3. SNPs linked to SLC34A1 are also associated with an increased risk to develop CKD in large GWAS data sets. The basis of this association remains to be elucidated. Nevertheless, their role in inherited or acquired forms of kidney disease is evident and these transporters may be interesting therapeutic targets for either gene or drug based therapeutic approaches.

## Acknowledgements

*None.*

### Financial support and sponsorship

*Work by the authors has been supported by the Swiss National Science Foundation.*

### Conflicts of interest

*C.A.W. has received honoraria by Kyowa Kirin and collaborates with Bayer AG.*

## REFERENCES AND RECOMMENDED READING

Papers of particular interest, published within the annual period of review, have been highlighted as:

▪ of special interest
▪▪ of outstanding interest
